# Bridging School and Practice? Barriers to the Integration of ‘Boundary Objects’ for Learning and Assessment in Clinical Nursing Education

**DOI:** 10.5334/pme.1103

**Published:** 2024-07-08

**Authors:** Malou Stoffels, Louti A. Broeksma, Margot Barry, Stephanie M. E. van der Burgt, Hester E. M. Daelmans, Saskia M. Peerdeman, Rashmi A. Kusurkar

**Affiliations:** 1 Amsterdam UMC, Vrije Universiteit Amsterdam, Faculty of Medicine, Research in Education, Amsterdam, The Netherlands; 2 Amsterdam UMC, VUmc Amstel Academy, Institute for Education and Training, The Netherlands; 3 LEARN! research institute for learning and education, Faculty of Psychology and Education, VU University Amsterdam, The Netherlands; 4 RadboudUmc Health Academy, Nijmegen, The Netherlands; 5 Amsterdam UMC location University of Amsterdam, Teaching and Learning Center (TLC), Amsterdam, the Netherlands; 6 Amsterdam Public Health, Quality of Care, Amsterdam, The Netherlands; 7 Amsterdam UMC, Vrije Universiteit Amsterdam, Faculty of Medicine, Department of skills training, The Netherlands; 8 Research institute: Amsterdam Public Health (APH), program Quality of Care, Amsterdam, The Netherlands

## Abstract

**Introduction:**

In clinical health professions education, portfolios, assignments and assessment standards are used to enhance learning. When these tools fulfill a bridging function between school and practice, they can be considered ‘boundary objects’. In the clinical setting, these tools may be experienced as time-consuming and lacking value. This study aimed to investigate the barriers to the integration of boundary objects for learning and assessment from a Cultural-Historical Activity Theory (CHAT) perspective in clinical nursing education.

**Methods:**

Nineteen interviews and five observations were conducted with team leads, clinical educators, supervisors, students, and teachers to obtain insight into intentions and use of boundary objects for learning and assessment. Boundary objects (assessment standards, assignments, feedback/reflection/patient care/development plan templates) were collected. The data collection and thematic analysis were guided by CHAT.

**Results:**

Barriers to the integration of boundary objects included: a) conflicting requirements in clinical competency monitoring and assessment, b) different application of analytical skills, and c) incomplete integration of boundary objects for self-regulated learning into supervision practice. These barriers were amplified by the simultaneous use of boundary objects for learning and assessment. Underlying contradictions included different objectives between school and practice, and tensions between the distribution of labor in the clinical setting and school’s rules.

**Discussion:**

School and practice have both convergent and divergent priorities around students’ clinical learning. Boundary objects can promote continuity in learning and increase students’ understanding of clinical practice. However, effective integration requires for flexible rules that allow for collaborative learning around patient care.

## Introduction

Learning in the clinical setting is crucial for health professions education (HPE), yet it is challenged by high workload, staff shortages as well as increasingly complex patient needs [1,2]. Schools and practice settings unite in their efforts to provide high-quality clinical placements. One way in which they collaborate is by providing resources and tools such as assessment forms, practice assignments and portfolios that fulfill a bridging function between school and practice [3,4,5,6,7,8]. Ironically, in practice, these resources and tools often have an adverse effect by creating extra work for students and supervisors while being experienced as irrelevant [9,10]. To date, the factors hindering the successful integration of these tools remain poorly understood [4]. The current study aimed to investigate barriers to the integration of tools for learning and assessment aiming to facilitate a bridge between school and clinical practice, as well as the underlying factors, in the context of clinical nursing education.

Varying types of tools are used to connect school and practice learning in HPE. Schools and practice settings share assessment standards to align the outcomes of different learning contexts [4,11,12]. Portfolios help students keep track of development, communicate their development with both school and clinical staff, and work towards predetermined outcomes across settings [8,13]. Mobile tools, e-learning modules, and reflection logbooks are used to connect learning in different contexts [4,6,14]. Although these tools and resources have different goals, they share the commonality of being utilized to support learning and assessment in clinical practice and being developed by, or in collaboration with, a school or university.

The literature suggests that the successful implementation of the same tools in different contexts is not easy: Ignoring local practice characteristics in developing guidelines for practice learning has been reported to hamper faculty’s creativity and personal judgement in postgraduate medical education [10] and create extra work for nursing students [9]. Assessment criteria may be used in different ways by clinical staff and school staff [15].

The inherent differences between schools and practice settings might cause challenges to use these shared tools in clinical practice. In practice settings, time and resource constraints make for prioritizing patient care over student education [16,17,18]. A focus on the completion of concrete, visible tasks in clinical practice can challenge the application of critical thinking and evidence-based practice skills taught in the school setting [19,20]. Even when clinical supervisors are committed to education, this is often frustrated by management’s focus on productivity [16]. Students’ temporary positions in the clinical team threaten the development of deep relationships and trust, which may threaten their safety to learn [21,22]. Clinical role models may demonstrate behavior that is misaligned with what the students learn in school [16,23].

Given the strength of practical learning experiences, training programs may not achieve their outcomes if students have limited opportunity to apply knowledge and skills in practice [24,25]. Overcoming these differences for successful collaboration between school and practice requires a shared underlying vision, training of all involved staff, communication about objectives, and time for implementation [4,6,15,26,27,28,29]. Although the literature indicates that inherent contradictions between school and practice present challenges at the organizational level as well as for individual students [30], a detailed investigation into how these contradictions affect integration of shared tools and standards for learning and assessment is lacking [4].

Two related frameworks that can be used to address learning at the intersection between different contexts are Cultural-Historical Activity Theory (CHAT) [31] and boundary crossing [3]. CHAT describes activity systems as a sum of interactions around an activity (e.g. becoming a doctor), or an organization, community of practice, or team with shared goals and activities (e.g. a family or a hospital) [32]. Each activity system is comprised of the interactions among the following: a) the individual subjects; b) their objects (objectives); c) the communities involved; d) the divisions of labor (tasks and responsibilities); e) the rules therein, and f) the mediating artifacts available. Overcoming tensions and contradictions within and between systems (such as the activity systems of university and clinical practice) can lead to learning and the transformation of practices [3,33]. When successfully resolving contradictions creates new forms of knowledge and activity, this is referred to as ‘expansive learning’ [34].

Rooted in CHAT, the boundary crossing perspective describes how people can learn and work at the boundaries of intersecting activity systems (such as school and practice or different disciplines) [3]. Tools and resources that are used and valued at the ‘boundary’ of two intersecting activity systems can be considered boundary objects [3,35]. Boundary objects are analytic concepts that play a role in different intersecting worlds (i.e., activity systems), and can adapt to the local needs of these worlds [36]. Boundary objects can be documents, technologies, or projects but also written sets of rules [37].

**Figure 1:**
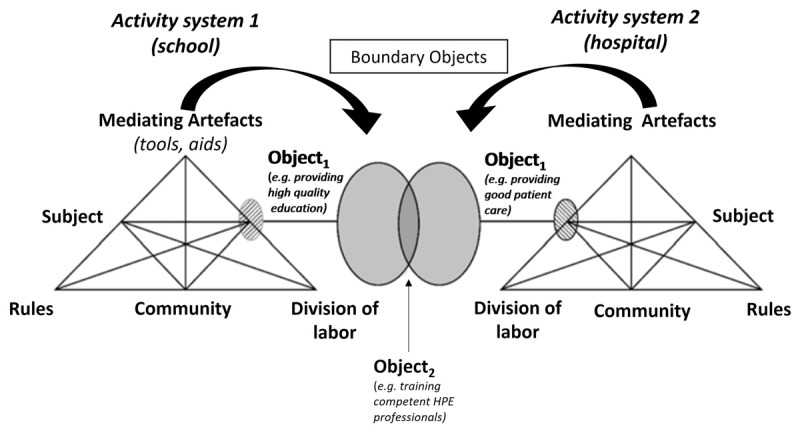
Activity systems with boundary objects. *Note*: Adapted from Moore, Ploettner and Deal [38]. *Object_1_* and object_2_ refer to *objectives* of the activity systems, whereas *boundary objects* refer to artefacts that are used to bridge between the two contexts.

See Figure 1 for an image of two intersecting activity systems, in which two separate systems (e.g., a university and a hospital) have individual objectives (object 1) as well as shared objectives (object 2) that can be achieved with the help of artefacts used in both contexts (boundary objects).

In HPE, CHAT has been used to unmask the contradictions that complicate clinical learning, as well as opportunities to resolve these contradictions. Within the clinical setting, students’ use of learning goals can create tension with the supervisors’ workload (division of labor) [33]. Learning to become a doctor while delivering safe patient care leads to tensions between roles (division of labor) and rules [39]. Competing objectives between departments can challenge collaboratively organized simulation training [40]. Tensions between rules and objectives can complicate workplace based assessment in the clinical setting [41]. CHAT was used to successfully *transform* practice by aligning the objectives of medical students and indigenous community members in placements [42], incorporating patients’ needs in a curriculum [43], and developing a placement model between universities. However, in HPE, contradictions *between the activity systems of school and practice* that affect learning in general and the use of shared resources and standards in particular, remain under-investigated.

In HPE, boundary objects between school and practice have been studied in the form of school buildings, [44] shared language in interdisciplinary working [45], feedback and prizes for clinical teachers [46], and simulation environments [47]. In the higher education literature outside HPE, portfolios [48,49], assessment standards [50], journals, reflection reports [51], videos [52] and digital tools [53] have been considered boundary objects. Boundary objects are used in interactions between people in different contexts and in cross-context activities [54]. Boundary objects can be developed both *for* and *in collaboration with* students [55]. Boundary objects are mostly designed within the school context, thereby limiting the inclusion of perspectives of the practice setting [54].

To summarize, tools that bridge between learning in school and practice can be considered ‘boundary objects for learning and assessment’. When these objects insufficiently adjust to the needs and characteristics of one of these activity systems [36], they fail to fulfill their bridging function. An analysis of the activity systems of school and practice can help understand barriers in the successful use of boundary objects in practice.

### Research question

What are barriers to the integration of boundary objects for learning and assessment in clinical nursing practice, and how can these be explained by contradictions between and within the activity systems of school and practice?

## Methods

### Study design

We conducted a qualitative study using different data collection methods and diverse types of participants in three phases.

### Setting

We conducted our study in the context of nursing education, where students spend about half of their training in clinical practice, spread across several clinical placements from 10–30 weeks. Because this context combines the power of hands-on learning with theory and skills instruction in the school setting throughout the training [26], it is well suited to answer our research question. Data were collected in an academic medical center within the Netherlands. The center offers clinical placements in collaboration with four different vocational institutions/universities of applied science, which educate for the same job description. All education tracks have competency-based curricula.

During their placement, students work alongside several clinical supervisors who are registered nurses with basic training in supervisory skills. Each nursing ward has a clinical educator to coordinate clinical training and a nursing team lead who is responsible for daily patient care delivery. During clinical placements, students remain in touch with school teachers/mentors during peer review meetings, assessment conversations, classes, or informally.

### Participants and sampling

The main researcher (MS) announced the study in two of the clinical educators’ team meetings. She followed this by emailing all clinical educators from one university hospital (n = 25) to ask about participation of their clinical wards. Of the 12 clinical wards whose clinical educators were willing to participate, students (n = 86), team leaders (n = 12), supervisors (n = 109), and allied school teachers (n = 8) were invited by email.

Inclusion was based on willingness. To keep the data manageable, we first invited participants up to a minimum number (three interviews in phase 1, eight in phase 2, and five observations in phase 3) per data collection phase (see Table 1). From those willing to participate, a purposeful sample was drawn aimed at maximum variation [56] in subpopulation and nursing ward, to include heterogeneity of perspectives and experiences. After the minimum numbers were reached, MS invited additional candidates until no new information relating to the research question was observed.

**Table 1:** Phases of data collection and aims.


PHASE	AIM	DATA COLLECTION METHODS

**1 Orientation**	Orientation on distinct categories of Boundary Objects, piloting interview	Pilot interview and first inventory for Boundary Objects with the clinical educator

		Screening of Boundary Objects that were sent after the pilot interview

**2 Intentions of boundary objects**	Insight into the types and nature of boundary objects (for follow up interviews and to inform analysis), insight into their intended objectives and use	Interviews and request to send additional Boundary Objects after the interview with clinical educators/team leads and school teachers

		Screening of Boundary Objects which were handed over after interviews

**3 Use of boundary objects**	Insight into experiences and barriers to the use of Boundary Objects	Interviews with supervisors^3^ and students, with Boundary Objects as primers

	Interactions around learning; role of Boundary Objects during an actual shift	Observations of students with their supervisors (*who did not participate in interviews*)


### Data collection

Data collection took place between April and June 2022. All data were collected by MS. See Table 1 for the distinct phases of data collection.

#### Collection of documents (phase 1 and 2)

In phase 1, MS conducted a pilot interview with one clinical educator to make an initial inventory of the boundary objects for learning and assessment, to inform further data collection and to pilot the interview guide (see supplementary file 1). The clinical educator was asked to send as many examples or pictures of boundary objects as possible to MS after the interview. An initial categorization of boundary objects was made. In phase 2, whenever a boundary object was referred to for the first time, the researcher asked whether they could provide a copy or picture of this object.

#### Interviews (phases 1, 2, and 3)

MS conducted semi-structured face-to-face interviews lasting between 37 and 65 minutes. One interview was held online. MS introduced and showed previously collected boundary objects and asked the participants for additional categories and examples. Questions were asked about the intentions behind these objects (phase 2 and 3) and actual experiences with their use (phase 3). Since an important characteristic of boundary objects is that they can adapt to local worlds [36], interviews aimed at understanding how boundary objects did or did not integrate into clinical practice. To understand which tensions within or between activity systems were underlying barriers to this integration, the interviews were guided by CHAT (see supplementary file 1 for interview guides). In line with the aim and scope of the study, the use of these boundary objects within the school setting was not explored in-depth. The interviews were audiotaped and transcribed verbatim by a research assistant.

#### Observations (phase 3)

MS shadowed students and peer students/supervisors for a full 8.5 hours shift. The observations focused on how the students shaped their learning process, how they interacted with others, and how they used or referred to boundary objects. Whenever possible, MS asked the student and supervisor to clarify behavior and perceptions. MS wrote extensive field notes immediately after each daily observation to create a thick description. A summary of the observations was sent to the participants shortly after the shift for approval.

### Analysis

We used the method of thematic analysis [57] informed by sensitizing concepts from activity theory [28,58]. In the first phase of data analysis, MS and LAB undertook in-depth reading of the transcribed interviews and observation field notes. They deductively coded all the transcripts with a coding tree including a) boundary objects b) objectives, experiences and barriers in the use of boundary objects c) components of the activity systems. The collected boundary objects were not analyzed separately but were consulted to interpret participants’ remarks. MAXQDA 2022 was used to aid data analysis [59].

Based on previous literature and our study aim [3,54], *boundary objects* were operationalized as ‘tools, aids and templates that fulfill a bridging function between school and practice to support learning and assessment’. We made a distinction between *structural* boundary objects, which were designed to use across boundaries for all students, approved by policy makers, and referred to in official guidelines, and incidental boundary objects which were used across contexts by groups or individuals but were not officially approved for these means. *Barriers* were operationalized as ‘negative perceptions about the intentions of a boundary object, its characteristics and/or effects, as well as difficulties in using them’. *Components of the activity systems* (subject, object, rules, division of labor, community, mediating artifacts) were coded whenever the participants referred to the characteristics of one of the two systems or when they became apparent during observations.

MS and LAB independently coded four (interview/observation) transcripts and discussed the findings. After adjusting code descriptions (e.g., clarifying the meaning of the components of the Activity Theory), MS and LAB discussed two more transcripts after which MS finalized coding of all the interviews.

MS made a summary of the different boundary objects including their use and function in both contexts. Next, MS categorized boundary objects and charted each category with barriers, experiences, and (conflicting) components of activity systems per type of participant that had come up in interviews and observations. Next, MS and LAB read through the data chart and revisited the transcripts to discuss what were the main barriers and how they could be described in terms of conflicting components within or between activity systems, and if and how they related to the type of boundary object, and type of participant.

MS inductively integrated these findings into four provisional overarching themes, supported by quotes that described how the barriers in the integration of boundary objects in clinical practice could be described in terms of conflicting components between and within activity systems. It should be noted that participants could represent both activity systems. For example, students alternate between school and practice periods, and teachers and practice educators collaborate and visit each other’s sites. Therefore, themes were not described in terms of diverse types of participants, but characteristics of the activity systems. MS and LAB reviewed these themes against the data, and refined them to make sure they were comprehensible and aligned with the research question, represented the data, and provided meaningful insights [57]. Finally, MS further described the themes and discussed with the team whether the descriptions were clear, whether quotations matched the descriptions, and whether the research question was answered until consensus had been reached.

### Reflexivity

We worked within an interpretivist approach, acknowledging that the constructions of reality are elicited through interactions between the researcher and participants [60]. To gain insight into the phenomenon of boundary objects as well as characteristics of the two activity systems, we collected data from different stakeholders and combined different data collection methods. During the analysis, we maintained a log of all the steps, which we discussed within the multidisciplinary team to make our choices and considerations explicit and justified and to manage personal or disciplinary bias. The Ethical Review Board of the Netherlands Association for Medical Education granted ethical approval (NVMO, file 2022.1.3). All participants provided informed consent.

## Results

We conducted 19 interviews (four clinical educators/team leads, three school teachers, seven students and five supervisors) and five observations. See supplementary file 2 for the participant characteristics.

We identified nine categories of boundary objects for learning and assessment. See supplementary file 3. for a summary of types of structural and incidental boundary objects, and their function and use in school and practice.

We identified three themes concerning the barriers to the integration of boundary objects for learning and assessment, as well as an overarching theme. We describe these themes in terms of underlying contradictions between and within the activity systems of school and practice below. Throughout the text, we refer to the different components of the activity systems (subject, object, tools, rules, community, division of labor) in italics. To avoid confusion with boundary objects, we have reworded the component *objects* to *objectives*.

### Conflicting requirements in the monitoring and assessment of clinical competency

School and practice learning was connected, by a shared competency framework, which described the competencies to be achieved after graduation, and the required competency level after each study year. This competency framework was translated into assessment forms and criteria which were used across the boundaries of school and practice. According to all participants, having this uniform standard, met the shared *objective* of monitoring and assessing students’ growth according to mutually agreed standards.

However, a couple of tensions affected the full integration and acceptance of this competency framework for both assessment and learning in clinical practice.

First, different priorities or nuances became apparent within the shared *objective* to educate nursing students: while the school trained students to become an all-round graduate that could work in different settings, for ward staff it was more important to prepare students for independent functioning on the ward they were posted. They both wanted to see this reflected in students’ assessment. This could challenge assessment decisions that had to be made in agreement between school teachers and clinical supervisors with the same boundary objects:

‘*We once had a discussion in which the school felt that the student should pass, and we all thought: no, that’s not allowed, and she passed anyway. Then, I think: that school looks in a different way than we do: how does someone function on a ward. And we did not think she functioned, so we thought she should fail, but the school thought she should pass, well then you miss the whole point.’ (supervisor 3)*

Not only were these standards used for formal assessments, but supervisors also wanted to use them to guide on-the-job decisions about what a student was or was not allowed to do. However, the abstract, uniform description of the competencies did not fit some characteristics of the clinical setting, such as the *objective* to ensure patient safety and the *community* with high staff turnover and large numbers of students:

‘*But sometimes I find it difficult … what students from different study years may or may not do [according to school’s criteria]. In my training, I used to have a sign-off list. Show it three times and then do it independently. So, I did ask the clinical educator a few times like, this task, is she allowed to do it? And then she says, for example, yes, it is very person-oriented sometimes. I find that difficult.’ (supervisor 5)*

Students selected patients and learning tasks on the ward that matched their competency development. However, the *division of labor* and *objective* to provide patient care did not always allow them to choose patients with the right complexity. Also, real patients didn’t always fit within the general, abstract descriptions within the competency framework, leaving students confused or frustrated when trying to apply this:

‘*…Sometimes you have a patient who is not that complicated in terms of pathology … But they ask an awful lot of you. Or, you know, just situations that arise. And then sometimes I find it difficult to indicate [using assessment criteria], okay, where do I stand? But also, for example you want to show your progression over the years. And of course, you do get more complex patients, and you become more independent. But sometimes you also have shifts in which it’s not like that. It feels like you must go higher and higher. But it’s not always like that.’ (student 3)*

### Different application of analytical skills

Another way in which theoretical and practice learning were bridged, was through boundary objects that encouraged students to critically analyze clinical practice and think about improvements. Examples included practice assignments, and stepwise approaches to clinical reasoning in which students can apply theory to actual cases. It should be noted that the latter were often not intentionally designed or established as boundary objects (i.e., incidental, not structural). However, students did use these, for example in practice assignments.

All types of participants applauded the *objective* of preparing students to become a deliberate practitioner and ultimately contribute to improvement of the quality of healthcare and acknowledged that school’s knowledge and tools could contribute to this objective. However, integration of these tools and templates in clinical practice was hampered by a couple of characteristics inherent to the clinical setting. For example, *distribution of labor* only allowed to spend limited time around each patient to meet the *objective* of producing fast, hands-on solutions. Students and supervisors wanted boundary objects such as templates to write a nursing plan to support these solutions, but found they did not:

‘*I often see that there is a bit of a gap: this is how I do it in my clinical placement and this is how I learned it, because at school you go so deep into it, that a lot of students think that writing a nursing plan implies working out point-by-point what you learned in school. Whereas here writing a nursing plan is: what is the patient suffering from now, … then the nursing plan is: tissue defect, the goal is to heal the wound and you only have to do that briefly … in school you go into it more in depth and more concrete and in practice, if you have to do that for every patient, then you don’t get to work.’ (supervisor 1)*

The differences in approach and objectives of clinical reasoning between school and practice meant that students perceived assignments, designed to connect practical experiences with theory as having little relevance:

‘*And then in school, you often reason about some bigger things or just, yeah, disease states or something…. To complete assignments, that’s what [these templates] are useful for. But for practice: yeah, a little less. I don’t really use it.’ (student 5)*

This lack of applicability was amplified by specific *rules* around school assignments on quality improvement. Although these rules met the shared objective of enhancing students’ ability to critically and systematically examine current healthcare practice, they hindered linking the assignments to relevant themes in the department:

‘*You may choose a case from the ward [in a practice assignment], but I chose a case study for ethics that had to do with corona and then all at once it was not allowed, because corona was above the law. Then I thought: it is something I really experienced on the ward, so that was quite a bit of a connection with the practice again [which I missed].’ (student 6)*

Thereby the assignments failed to meet the *objective* to let students experience the value this analytical and critical reflection on healthcare, which was shared among all stakeholders. Moreover, the *rule* of completing assignments individually did not fit well with the *objective* and division of labor to work as a team.

Likewise, the observations showed that while providing patient care, the students spontaneously consulted information resources from the ward, but not from school. This exemplifies that the prevalent boundary objects are not easily integrated into the daily work process *(division of labor)* within the ward.

### Difficulty to integrate boundary objects for self-regulated learning into daily supervision practice

To make sure students developed the skills to self-regulate their learning process, students were taught to write development plans, formulate learning goals, and self-assess their progress. The school provided them with training to do so and with templates such as for development plans to be used during placements. Both teachers, students and clinical staff supported the *objective* of preparing students to take the lead in their learning process.

However, several aspects of current boundary objects for self-regulated learning do not align well with the characteristics of the ward. The school’s goal of supporting student learning in a structured manner resulted in extensive templates for written plans. Supervisors saved time to prebrief and brief students’ shifts to discuss their learning. However, the *division of labor* on a ward did not allow supervisors to include students’ written plans into these discussions:

‘*They do send their development plans to us. But we ourselves don’t give direct feedback on it. The clinical educator does that. We must go through it. But we don’t really have a lot of time for that. So, I see it come along sometimes, but I don’t have a clear picture of it.’ (supervisor 5)*

Moreover, some supervisors questioned whether the learning goals that students had to derive from their development plans helped meet *objectives* within clinical practice:

‘*Yes, to achieve their goals. That’s what learning goals contribute to. But I don’t think it necessarily makes them a better nurse. Because a nurse who doesn’t have a learning goal, they have to achieve the same goals. This might only make them more aware of what they need to achieve. And that maybe at the end they won’t be in for a surprise.’ (PE/team lead 1)*

Thereby, the plans and goals that students formulated, became detached from their daily interactions with supervisors. As a school teacher commented, this lack of supervisor involvement would undermine the value and eventual use of the plan:

‘*[To motivate students…] would require that supervisors start using that development plan, where are you, what do you want to develop… also do the role modeling part more explicitly. So that a student also doesn’t feel like they didn’t make their development plan for nothing.’ (gg 2)*

Likewise, students commented that the way they had to assess themselves *(rules)* was lacking in transparency and too complex for supervisors to critically respond to. Consequently, by writing this they missed important input from their supervisor about their progress:

‘*After all, it’s your feeling, isn’t it? Because you have to fill in the crosses [of the self-assessment template] yourself. And your supervisor is like, yeah, fine. And based on where you think you are, you submit that. And it might just be that the supervisor thinks, well, that’s pretty much right … And there’s not really any insight of their own in return.’ (student 1)*

### Learning within an assessment-driven system amplifies barriers

As an overarching theme, we found that the lack of adaptation of boundary objects to the local context was amplified by the intertwining of learning with assessment: feedback and written reflections were used to demonstrate competency, and assignments and plans received grading form the school. This drew students’ attention away form making boundary objects useful for learning in the clinical setting. In fact, this also highlighted a tension between the *objective* of learning and the *objective* of assessment *within* both activity systems.

For example, the learning potential of boundary objects such as development plans, was undermined because students used them for the purpose of checking off tasks whereas the school teachers believed their purpose was to stimulate reflection:

‘*Sometimes [the development plan] becomes a checklist. I wrote this down as indicators like, I need to show that I can insert a catheter at least once. Okay, check. But I think, okay, during this task you may well have had conversations with the patient. Or there was an emergency next door. How did you act? That you broaden that reflective capacity rather than just that one little point.’ (teacher 3)*

Moreover, the fact that students felt they constantly have to ‘prove’ their level of competency resulted in competency standards getting in the way of learning instead of supporting learning:

In observation number 1 the student repeatedly received negative feedback and appeared to have little overview of her patients during the shift. Her performance seemed to worsen throughout her shift, and she proclaimed having stress and feeling bad about it. At the end of the shift, her supervisor advised her to take a smaller patient load for the next shift, but the student exclaimed in tears that if she did not have a patient load of three, she would not meet assessment criteria by the end of her clinical placement.

Conversely, other students took advantage of the ambiguity surrounding assessment criteria by manipulating the assessment criteria to their liking.

‘*I almost always bet high because then I pass. I think I’m performing well and if not, they’ll let me know, and I’m not going to burn my fingers. This week someone said: “Why do you put your crosses [indicators of competence in feedback forms] so high?” I said: “yes why not, as long I pass.” (student 6)*

Some assignments received separate grades from the school. This met the school’s *objective* to value students’ individual achievement and maintain quality. The students reported how this grading as well as the *rules* to achieve satisfactory grading decreased their motivation to connect the assignments to the requirements of the wards:

One student (observation 2) reported that one of the practice assignments was to make an improvement plan for the ward. She had had a great idea, which was confirmed by surveys with the ward staff. When the observer asked whether the idea had in fact been implemented on the ward, the student shook her head indifferently, and replied when asked: ‘*no, because the assignment did not require this*’.

This suggests that grading and assessment further impede the integration of boundary objects into working processes on the clinical ward.

## Discussion

The present study identified the barriers to the integration of boundary objects for learning and assessment in the context of clinical nursing education. These barriers related to conflicting requirements from school and practice to clinical competency standards and analytical skills, as well as poor integration of boundary objects into student-supervisor interactions around the learning process. These barriers were amplified by the simultaneous use of boundary objects for learning and assessment. Our analysis guided by CHAT suggested that boundary objects can contribute to the shared objective of assuring competency development across settings, assisting students to actively guide their learning, and encouraging analytic and critical thinking to better understand healthcare practice and reflect on possible improvements. However, school and practice also have divergent objectives or priorities: the school’s objective is to provide a broad, transferable education, and practice’s priority is to make sure students can contribute to patient care on the ward. These different objectives became visible when the rules around boundary objects hampered the successful integration of boundary objects within the community (changing workforce) and distribution of labor (collaborative patient care combined with training students) in the clinical setting. The reported barriers to integration might result in a loss of time, energy, and chances for schools and practical experiences to mutually strengthen each other.

The contradiction between working and learning identified in previous studies [16,17,31] was partly reflected in the current findings. For example, the urgency of patient care did not allow students to extensively analyze or prepare clinical cases. The current study added contradicting perspectives on *how* to spend time in learning clinical practice. For example, the supervisors were willing to grant students time to work on school assignments, if they could see how they would *ultimately* contribute to the objective of better patient care. Likewise, supervisors were willing to invest time in discussing students’ learning goals, but as part of the oral pre-shift briefing. This suggests that boundary objects that conflict less with the community and division of labor in the clinical wards (high workload and staff turnover [61], collaboration [18,62], and flexibility [63]) could help achieve shared objectives between school and practice. On the one hand, this requires boundary objects that can be integrated into clinical work, which can directly support the delivery of patient care, ‘just-in-time learning’, co-regulated learning and reflection [62,64,65,66,67]. Faculty training should inform supervisors how to allow and support students to use these boundary objects. On the other hand, this requires boundary objects which can be used in off-time to collaboratively discuss and improve patient care around meaningful issues facing the ward [68]. Future studies should further inform the nature and quality criteria of these objects.

In line with the literature on how assessment can diminish students’ intrinsic motivation for learning and teamwork [16,41,69], the current findings show tensions between the objectives of learning and assessment. Additionally, the study highlights several ways in which learning can be further compromised when assessment standards are used across boundaries: uniform and abstract assessment criteria can make students select irrelevant or inappropriate learning opportunities, and can make them engage in strategic behavior to demonstrate competency. These translation difficulties suggest that it may not be entirely feasible to use the same assessment criteria across settings. One development that might help align students’ learning with the ward’s needs, is the transition to Entrustable professional activities (EPA’s). EPA based curricula describe the outcomes of learning in terms of concrete, recognizable activities, yet allow practice settings to teach and assess these activities in a way that aligns with the local way of healthcare delivery [70]. Future studies should explore whether defining assessment standards in terms of EPAs might help align the goals of reliable, uniform assessment with the objectives and division of labor of the ward.

According to the tenets of CHAT, the identification of contradictions within and between activity systems can lead to enhanced learning and/or change at the individual or system level when these contradictions are successfully overcome [71]. The current findings indeed provide examples of this ‘expansive learning’, where both students and supervisors learn from students’ critical analysis of patient care with the help of practice assignments provided by the school. However, the data also suggest that contradictions between activity systems often remain unresolved. For example, although students wanted to contribute to the ward, they were fulfilling their practice assignments in a way that did not feel relevant but would lead to a good grade. This suggests that the complexity of the intersecting activity systems leads to tensions that students are not able to resolve within their vulnerable and temporary position as learners [41]. This is troublesome as trying to navigate incompatible demands may cause moral distress to students and frustrate their identity development [30]. Therefore, it is important to collaboratively revisit the structures that give rise to these tensions and when necessary revisit the ideology of learning in practice [29,30]. This ideology can further inform the design and use of boundary objects and make their purpose more clear for all stakeholders [26].

There are several limitations to the present study. First, it was conducted with a single discipline in a single hospital, with an established tradition of education and training. The voluntary nature of the study may have attracted participants that were particularly engaged in education [72]. This ensured that most participants were familiar with and supportive of boundary objects but may have limited transferability to other settings. However, describing the barriers in terms of inherent characteristics of the clinical and academic setting informed by CHAT can help educators understand the same challenges in different contexts. An even more comprehensive understanding of boundary objects would require a more thorough exploration of their integration within the school setting. Unlike studies targeting a specific boundary object such as a portfolios or learning goals program [8,33], we simultaneously looked at different objects with different purposes, without performing a control of their quality. Because these objects have the shared purpose of supporting learning and assessment across settings, we consider the choice to classify all these as boundary objects a strength of the study. An additional strength of the study is the inclusion of diverse types of participants and diverse types of data collection.

### Implications

Schools and practice settings should collaborate in developing boundary objects that can optimize students’ learning process within the rich clinical setting and use authentic learning situations to develop students into, lifelong learners who adopt an analytical attitude toward healthcare. This requires a shared underlying ideology of the principles of learning in practice. Hospitals should receive the freedom to apply or adjust boundary objects in a way that suits the work processes and culture of the ward. Policy makers and educators should be aware of the potential consequences of grading and detailed criteria on students’ learning process.

### Conclusions

School and practice have different priorities and resources, but they share the objective of creating a future healthcare workforce. Boundary objects such as assignments, development plans and assessment standards can promote continuity in learning, support students in adopting an analytical and critical attitude to practice, and help them achieve a broad set of competencies. A focus on individual performance, detailed and uniform criteria, and assessment hampers the integration of these boundary objects into daily working and learning processes on the ward. Redesigning boundary objects while keeping motivational principles in mind [69] could result in capitalizing on the inherently different qualities of the schools and practice settings.

## Additional Files

The additional files for this article can be found as follows:

10.5334/pme.1103.s1 Supplementary File 1. Interview Guides.

10.5334/pme.1103.s2 Supplementary File 2. Participant characteristics per data collection phase.

10.5334/pme.1103.s3 Supplementary File 3. Types of boundary objects for learning and assessment and function and use in school and practice.
