# Supplementary material for: Bridging School and Practice? Barriers to the Integration of ‘Boundary Objects’ for Learning and Assessment in Clinical Nursing Education
Source: Perspect Med Educ. 2024 Jul 8;13(1):392–405. doi: 10.5334/pme.1103 (PMC11243767; doi:10.5334/pme.1103)
Supplement: Supplementary File 1. — Interview Guides. [file pme-13-1-1103-s1.pdf]

Supplementary file 1: interview guides

In this interview, we will discuss nursing placements. School and practice have together or individually developed tools, aids and standards to help students structure their learning, link theory to practice, and learn across all those internships, toward an eventual final level.

In this study, we are interested in how these tools work out in practice, and when they are experienced as helpful, and when not.

We will use this data anonymously to eventually make recommendations about how to optimize placement learning. We are interested in your personal views and experiences. There are no right or wrong answers. If you do not know something or do not have an opinion about something, that is totally okay. I would like you to sign a consent form.

Can you start by telling a little bit about yourself?

I would like to introduce some types of tools, aids and formats that fulfill a bridging function between school and practice to support learning and assessment. we will discuss them later in detail.

- Standards and formats that can be used during patient care (for example stepwise approaches for clinical reasoning, or for taking a patient history),
- competency (self-assessment) assessment rubrics,
- personal development plan formats,
- feedback formats
- practice assignments
- skill assessment forms
- reflection forms
- regulations in which these are described; information provided about expectations

Are these familiar to you? do you have any additions?

Questions to be asked per type of boundary object[1]:

- *"intentions of boundary objects" interviews:* can you name objects in this category and send them afterwards?. *"use of boundary objects"* i*nterviews*: which of these objects (shown by the interviewer) do you know or use?

[1] as interviews are semi-structured, questions are not necessarily asked in the same order. The interviewer tries to gather above mentioned information while connecting to the interviewee's narrative as much as possible

- *"intentions of boundary objects" interviews:* why is this object used? Are there different purposes? Do you think these purposes are met? *"use of boundary objects"* i*nterviews*: What do you think the purpose of this object is? Do you think this purpose is met?
- What (different) rules and arrangements around the use of this object exist?
- How, when and by who is this object used? is it used in different contexts?
- Are there similar objects, and how does their use co-occur?
- How does it fit within working processes on the ward and/or in school?
- *"use of boundary objects"* i*nterviews*: How do you experience the use of this object? Do you experience any challenges around their use?
- *"intentions of boundary objects" interviews:* How has this object been implemented?

Wrap up questions:

- *"intentions of boundary objects" interviews:* what else do you do to support students bridge between theory and practice?
- *"use of boundary objects"* i*nterviews:* How do you generally experience support to bridge between theory and practice?
- Do you have anything else to discuss?
