# Supplementary material for: Bridging School and Practice? Barriers to the Integration of ‘Boundary Objects’ for Learning and Assessment in Clinical Nursing Education
Source: Perspect Med Educ. 2024 Jul 8;13(1):392–405. doi: 10.5334/pme.1103 (PMC11243767; doi:10.5334/pme.1103)
Supplement: Supplementary File 2. — Participant characteristics per data collection phase. [file pme-13-1-1103-s2.pdf]

Supplementary file 2. participant characteristics per data collection phase.

Data collection phase	Code	Role	Current ward	Current Higher education institute (HEI)	Gender
2	CE/TEAM lead 1	Clinical educator (with supervisor tasks)	internal medicine		F
2	CE/TEAM lead 2	Clinical educator (with supervisor tasks)	neurology		F
2	CE/TEAM lead 3	Clinical educator and team lead (dual role)	hematology		F
2	CE/TEAM lead 4	Team lead	nephrology		F
2	Teacher 1	Teacher	Teacher	HEI 2	M
2	Teacher 2	Teacher	Teacher	HEI 1	M
2	Teacher 3	Teacher	Teacher	HEI 1	F
3	Student interview 1	student (interview)	obstetrics	HEI3	F
3	Student interview 2	student interview	nephrology	HEI 1	F
3	Student interview 3	student interview	oncological surgery	HEI 1	F
3	Student interview 4	student interview	acute admissions ward	HEI 1	F
3	Student interview 5	student interview	oncological surgery	HEI 1	F
3	Student interview 6	student interview	neurology	HEI 1	F
3	Student interview 7	student interview	short stay	HEI 1	F
3	Supervisor interview 1	Supervisor (interview)	nephrology		F
3	Supervisorinterview 2	Supervisor (interview)	oncological surgery		M
3	Supervisor interview 3	Supervisor (interview)	Ear, nose & throat		M
3	Supervisor interview 4	Supervisor (interview)	internal medicine		F
3	Supervisor interview 5	Supervisor (interview)	acute admissions ward		F
3	Observation 1	student + supervisor (observation)	oncological surgery	HEI3 (student)	F + F
3	Observation 2	student + supervisor (observation)	pulmonology	HEI4 (student)	F + F
3	Observation 3	student + supervisor (observation)	gastroenterolog	HEI 1 (student)	F + F
3	Observation 4	student + supervisor (observation)	obstetrics	HEI3 (student)	F + M
3	Observation 5	student + supervisor (observation)	neurology	HEI 1 (student)	F + F

Note: At the time of the study, Higher education institute 1 dominated the student population in the hospital. However, to reflect the reality of wards with a mix of different studies, we decided to include students and teachers from the other institutes as well.
