# Supplementary material for: Bridging School and Practice? Barriers to the Integration of ‘Boundary Objects’ for Learning and Assessment in Clinical Nursing Education
Source: Perspect Med Educ. 2024 Jul 8;13(1):392–405. doi: 10.5334/pme.1103 (PMC11243767; doi:10.5334/pme.1103)
Supplement: Supplementary File 3. — Types of boundary objects for learning and assessment and function and use in school and practice. [file pme-13-1-1103-s3.pdf]

Supplementary 3. Types of boundary objects for learning and assessment and function and use in school and practice

Note: when variation around the use of boundary objects was found (between schools, study years, wards, tracks), this is marked in italics

<table>
<tr><th></th><th>Boundary object</th><th>Function and use in school</th><th>Function and use in practice</th></tr>
<tr><td rowspan="3">Standards and formats that can be used during patient care</td><td>Formats for clinical reasoning</td><td rowspan="3">• Teachers offer training and case-based practice around the formats and provide them to students</td><td rowspan="3">• Students can apply formats to guide learning and working in clinical practice when required.
• Sometimes clinical educators adapt or extend formats for students to make them more applicable</td></tr>
<tr><td>Formats for communication</td></tr>
<tr><td>Formats/ handouts for preparing patient care, writing nursing plans, taking a patient history</td></tr>
<tr><td>Theory resources</td><td>Textbooks, PowerPoints, movie clips etc. about nursing theory</td><td>• School teachers provide resources during classes so students can apply theory to clinical practice</td><td>• Students can consult materials in clinical practice and discuss them with supervisors</td></tr>
<tr><td rowspan="2">Competency (self-assessment) rubrics</td><td>Self-assessment forms</td><td rowspan="2">• Schools offer self-assessment rubrics and provide students information about these
• Schools offer information, handouts etc. to practice settings about their assessment criteria
• Schools provide rules about when and how to fill in self-assessments
• Schools provide assessment standards including norms per study year and determine pass/fail rules
• School teachers make assessment decisions together with practice representatives</td><td rowspan="2">• Students prepare their self-assessments based on 'evidence' of their clinical competencies
• Students send their self-assessments to supervisors, clinical educators and school teachers
• Supervisors and clinical educators fill in assessment forms
• Supervisors and clinical educators make assessment decisions together with school teachers
• Clinical educators sometimes make posters, handouts, to make competency criteria more understandable, or provide opportunities in which can collaboratively work on applying competencies to patient care on the ward</td></tr>
<tr><td>Assessment forms</td></tr>
</table>

**Skill assessment forms**		• *In some education tracks, the school requires students to have specific skills assessed*	• *In some education tracks, supervisors/clinical educators fill in skill assessment forms*<br>• *On some wards, clinical educators develop additional skill assessment forms*
**Personal development plan formats**	Format in which students describe how they are going to achieve the required competencies, at the beginning of each placement	• School teachers provide formats and give instruction about these<br>• Schools set rules and deadlines around development plan formats<br>• *Schools give feedback and/or grade development plans (in some tracks)*	• Students discuss plans with supervisors/ clinical educators<br>• Students use learning goals from their plans at the beginning of the shifts<br>• Students evaluate goals from their development plan in assessment meetings<br>• *Some clinical educators create visual spaces (e.g. White boards) in which students' learning goals become visible for all ward staff*
**Feedback formats**	Formats to collect daily, weekly monthly feedback	• Schools provide feedback formats and set rough guidelines about how often to collect feedback and how to use this feedback for (self) assessment	• Student writes self-evaluation after the shift<br>• Student collects written feedback with the formats and writes self-evaluations in the formats<br>• Students use feedback as evidence for their competency achievement<br>• Supervisors and clinical educators use feedback forms to keep track of development<br>• *Practice educators sometimes set additional guidelines for the frequency of feedback forms*
**Practice assignments**	Assignments or projects for students to elaborate on clinical cases, make quality improvement plans, analyze nursing care using Evidence Based Practice etc.	• Schools provide training and give instructions about assignments<br>• School teachers grade assignments	• Students discuss with supervisors/clinical educators how they can apply assignments to practice<br>• Supervisors/ clinical educators provide feedback on assignments<br>• *Students sometimes present outcomes in*

			ward meetings
**Reflection formats**	Formats for stepwise reflection	• Schools provide training about reflection and offer formats for written reflection<br>• *Schools (in some educational tracks) require students to include written reflections to demonstrate competency achievement*	• Students discuss clinical situations to reflect on with supervisors/ clinical educators<br>• Students receive feedback on written reflections
**Clinical placement guidelines**	Guidelines in which expectations concerning the use of these boundary objects for learning and assessment are described	• Schools provide general guidelines about the use of boundary objects in clinical practice including the rationale behind them	• Clinical educators provide additional guidelines about how to use and apply these formats to the particular setting
